# Sickness Presenteeism in Shift and Non-Shift Nurses: Using the Fifth Korean Working Conditions Survey

**DOI:** 10.3390/ijerph18063236

**Published:** 2021-03-21

**Authors:** Ari Min, Minkyung Kang, Hye Chong Hong

**Affiliations:** 1 Department of Nursing, Chung-Ang University, Seoul 06974, Korea; amin@cau.ac.kr (A.M.); julieh@cau.ac.kr (H.C.H.); 2 College of Nursing, Keimyung University, Daegu 42601, Korea

**Keywords:** presenteeism, nurses, shift work schedule, sleep disturbance, health problem

## Abstract

Nurses have reported higher rates of sickness presenteeism than other workers, which is particularly problematic because this problem is linked to care quality and patient safety. This secondary data analysis study aimed to identify the prevalence of sickness presenteeism and explore related factors among shift and non-shift nurses using the Fifth Korean Working Conditions Survey. A total of 272 nurses in Korean hospitals were included. The survey included questions on working conditions, health status, and sickness presenteeism. A multivariate logistic regression was used to identify associated factors of sickness presenteeism. Overall, 21.8% of the participants reported experiencing sickness presenteeism; shift nurses experienced more sickness presenteeism than non-shift nurses. Sickness presenteeism was greater in shift nurses who did not have rest breaks during work and in nurses who experienced quick return. Additionally, the odds of sickness presenteeism were approximately four times greater in shift nurses who experienced sleep disturbance and about four times higher in shift nurses who experienced health problems. Among non-shift nurses, the odds of sickness presenteeism were about 15 times greater in those who worked ≥53 h per week. Nurse managers and administrators should prevent sickness presenteeism in hospital nurses to provide quality care and enhance productivity.

## 1. Introduction

Sickness presenteeism (SP) is defined as being present at work when feeling sick and unable to fully perform in the workplace [1,2,3]. This concept has received considerable attention in the field of occupational health [4] because it negatively affects work performance, lowers work productivity [2,5], and increases financial burden [6,7,8]. The prevalence of SP has been reported to be approximately 35% in European countries [9] and 23% in Korea [10].

Nurses have been reported to exhibit higher rates of SP than other workers [1,3]; this is particularly problematic because SP in hospital nurses is linked to care quality and patient safety [4,11]. Healthcare professionals generally have a strong sense of duty, which can pressure them to attend work despite illness, especially during staffing shortages [8]. Particularly, South Korea faces the largest nurse shortage compared to previous years, as turnover rate is considerably high at 15.4% overall and 45.5% in newly graduated nurses, leading to high nurse-to-patient ratios and increased workload [12]. Nurses who attend work while ill cannot function at their full capacity, increasing the likelihood of negative patient outcomes, such as falls and medication errors [5,11,13,14]. Thus, it is important to identify the factors that can reduce SP among nurses.

Recent research on the predictors of SP indicates that work-related factors have a greater impact than individual characteristics (e.g., gender, age, occupation, and education) [15,16,17]. Work-related factors associated with SP include shift work, job demand, workload, understaffing, long working hours, overtime, job satisfaction, job insecurity, and relationships with colleagues [9,15,18,19]. Notably, shift nurses are particularly vulnerable to long hours and insufficient rest [20,21,22]. For example, in a recent study of Korean nurses, approximately 70% of shift nurses worked over 40 h per week, including about 10 h of overtime per week. Furthermore, a majority of them (96%) did not have guaranteed regular breaks during work hours [22]. Research indicates that these factors can lead to SP among workers in other industries [19,23]. However, despite the importance of SP in this population, to the best of our knowledge, no nursing studies have investigated the relationship between working hour characteristics and SP. 

Health and sleep problems are strong risk factors for SP in various occupations [16,24,25]. To ensure high-quality care and reduce preventable medical errors, healthcare systems must prioritize the good health of nurses on duty [14]. Common health problems among nurses include musculoskeletal pain, migraines, stress, depression, anxiety, and low sleep quality, which can lead to increased SP [13,17,26,27]. However, despite differences such as working hour characteristics and health status between shift and non-shift nurses, previous studies have not accounted for shift work. Therefore, there is a dearth of knowledge on whether health-related problems would increase the risk of SP in shift nurses, non-shift nurses, or both. 

In Korea, whether a nurse works shifts or not is commonly arranged according to their working unit. For instance, the majority of nurses working in outpatient units, operating departments, and treatment rooms worked fixed day shifts (e.g., working from 9 a.m. to 6 p.m.), while nurses working in general wards, intensive care units, and emergency rooms worked in rotating shifts including night shifts. The most common pattern of work among shift nurses is eight-hour shifts with the shift changing every two to three days [28]. Although studies reported that shift work increases the risk of SP among general workers [9], the evidence may not be generalizable to nurses who work in rotating shifts. Understanding the associations of working hour characteristics and nurses’ health with SP would be beneficial to implementing efficient management strategies to reduce SP among hospital nurses. Therefore, this study aimed to determine the prevalence of SP and to examine the associations among working conditions, health status, and SP among shift and non-shift nurses in South Korean hospitals.

## 2. Materials and Methods

### 2.1. Data Source and Sample

**Figure 1:**
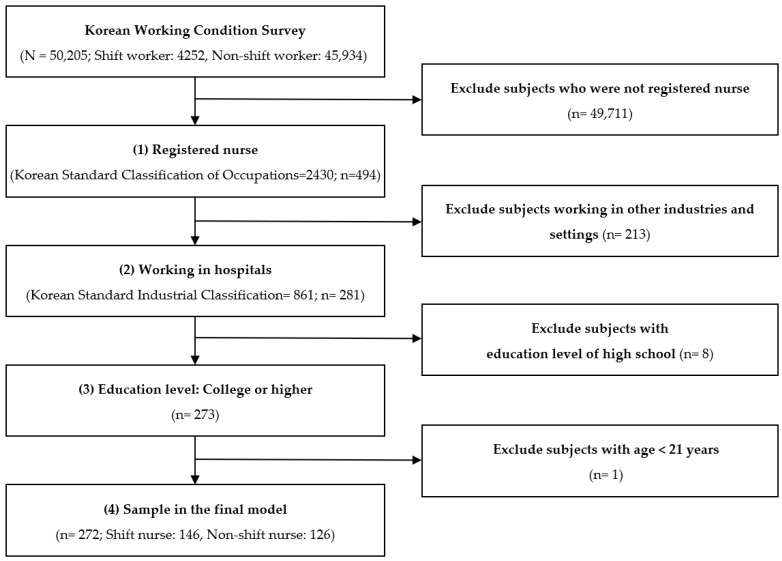
Flow chart of study population.

This study was a secondary analysis of the Fifth Korean Working Conditions Survey (KWCS) [29], and an institutional review board determined the study to be exempt (No. 1041078-202006-HRSB-147-01). The Fifth KWCS used a cross-sectional design and collected data between 11 July and 17 November 2017 in 17 cities and provinces nationwide [30]. The survey first extracted stratified districts and then extracted households, using probability proportional to size systematic sampling. Professional investigators visited the selected households and interviewed one individual worker each, aged over 15. The eligible individuals were workers (either employees or self-employed) who worked at least one hour for pay and profit during the last week at the time of the interview. Retired and unemployed individuals, housewives, and students were excluded. The participants were informed of the voluntary nature of the participation and confidentiality of their responses. The total number of households for interviews was 130,645, and the total number of survey respondents was 50,205. Households were replaced if the investigators could not interview eligible individuals after visiting four times. Households were excluded for (1) refusal: 28,262 cases, (2) non-contact: 8808 cases, (3) inability to interview (e.g., drinking, speech impairment): 2194 cases, (4) ineligible: 15,032 cases, (5) unknown eligibility: 23,007 cases, and (6) unknown residence: 3137 cases. The survey response, cooperation, and refusal rates were 44.9%, 64.0%, and 25.3%, respectively. In this secondary analysis, the inclusion criteria were (1) being a registered nurse and (2) working in hospitals (Figure 1). Respondents who reported their educational level as high school were excluded because all registered nurses in South Korea must have an associate degree or higher. Finally, 272 nurses were included. 

### 2.2. Validity and Reliability

The KWCS was developed based on the European Working Conditions Survey (EWCS) to provide employees with better working environments by identifying work-related factors and their health effects. The validity and reliability of the KWCS were assessed in a previous study [30]. The external validity was assured using sound sampling procedures (i.e., multi-stage stratified cluster random sampling). The content validity was guaranteed through a rigorous translation and back-translation process. Specifically, the content validity was also assessed by expert review focusing on conceptual and functional equivalence. For example, it was necessary to recheck the concepts of “business”, “place”, “site”, and “establishment” in the EWCS with the EWCS questionnaire developer to clarify their translation in Korean in the process of validating the Fifth KWCS [31]. After conducting the pretest, including real-life (e.g., interviewer–respondent interaction and technical functioning of the questionnaire) and cognitive interviews, some alterations, such as revising the questionnaire structure, adding additional instructions, and rephrasing questions, were made based on the pretest results. The results obtained using the test–retest method (i.e., a telephone survey of 30% of the total respondents) demonstrated the high reliability of the KWCS (i.e., matching rates of questions ranged from 98.5% to 99.6%). Moreover, a consistently high level of reliability can be guaranteed through the sophisticated procedures used in the field survey and the technical manual provided to the interviewers. 

### 2.3. Study Variables

#### 2.3.1. Sickness Presenteeism

SP was measured by asking, “Over the past 12 months, did you work when you were sick?” Participants who reported “yes” were considered to have experienced SP.

#### 2.3.2. Working Conditions

The study included three working hour characteristic variables: total working hours, rest breaks during work, and quick return (i.e., rest period between shifts < 11 h). The participants reported their total weekly working hours, which were divided into <40, 40−52, and ≥53. The categorization was based on the Labor Standards Act, which states that working hours can range from 40 to a maximum of 52 h per week [32]. Rest breaks during work were assessed by asking the participants if they were able to rest when desired. There were five possible responses: “always”, “most of the time”, “sometimes”, “rarely”, and “never”. Responses of “always”, “most of the time”, and “sometimes” were categorized as “yes”, while those of “rarely” and “never” were categorized as “no”. [4] The participants were also asked, “Have you had <11 h between the end of one working day and the start of the next working day at least once?” The response options were “yes” or “no”.

#### 2.3.3. Health Status

Nurses’ health status was assessed based on sleep disturbance and health problems. Sleep disturbance was measured by asking, “Over the last 12 months, how often did you have any of the following sleep-related problems?” The response options were “daily”, “several times a week”, “several times a month”, “not very often”, and “never” for each sleep-related problem: (1) difficulty falling asleep, (2) waking up repeatedly, and (3) waking up feeling exhausted and fatigued. For each sleep-related problem, responses of “daily”, “several times a week”, and “several times a month” were categorized as “yes”, while those of “not very often” and “never” were categorized as “no”. [33] Participants who responded “yes” to at least one problem were considered to have experienced sleep disturbance.

Participants who responded “yes” to at least one of the following health problems over the preceding 12 months were considered to have a health problem: hearing problems; skin problems; backache; shoulder, neck, and upper limb pain; lower limb pain; headaches; eyestrain; injury; anxiety; overall fatigue; and others. 

#### 2.3.4. Demographic Variables

Demographic variables included age, gender, educational level (associate/bachelor’s/master’s degree or higher), working experience, and location (urban/rural). Age was categorized as <30, 30−39, 40−49, or ≥50 years. Working experience as a registered nurse in the current hospital was categorized as <1 year, 1−4 years, 5−9 years, or ≥10 years. 

### 2.4. Data Analysis

We analyzed the data using STATA 15.1 (StataCorp LP, College Station, TX, USA). The KWCS provided a weight to adjust for potential bias of the sampling process (i.e., sample design, non-response, and post-stratification) and recommended employing weighting adjustment in data analyses. The weighting was carried out based on participant distribution by region, city size, gender, age, economic activity, and occupation to make the population structure for the survey similar to the population structure of Korea. Therefore, we performed weighted statistical analysis in this study. To investigate the relationship between the demographics, working conditions, health status, and SP by shift type, a chi-square test was conducted using the STATA command “svy: tabulate”. We analyzed the associations of working hour characteristics and health status with SP using multivariate logistic regression models, controlling for demographics (age, educational level, work experience, and location) with the STATA command “svy: logit”. Statistical significance was set at *p* < 0.05, 0.01, and 0.001 for all analyses.

## 3. Results

The participants’ characteristics are summarized in Table 1. There was no statistical difference in demographics, working conditions, and health status between shift and non-shift nurses. The proportion of shift and non-shift nurses was 55.7% and 44.3%, respectively. The average age was 37.33 (±8.69) years, and those aged <30 years accounted for 42.5% of the sample. Most nurses were female (95.2%) and had a bachelor’s degree (68.5%). The mean working experience in the current hospital was 8.63 (±11.66) years, and 51.5% of the hospitals were in rural areas. More than two-thirds (66.8%) had worked <40 h during the preceding week, and 59.7% had rest breaks during work. Approximately 8% of nurses reported having experienced <11 h between the end of one working day and the start of the next at least once in the previous month. Approximately 20% of nurses had experienced sleep disturbances, and 33% experienced health problems. 

Of the nurses, 21.8% reported experiencing SP in the preceding 12 months. The rate of SP was higher among shift nurses (*n* = 35, weighted percentage 54.6%) than non-shift nurses (*n* = 28, weighted percentage 45.4%). Among shift nurses, only quick return, sleep disturbance, and health problems were significantly associated with SP (Table 2). The SP rate was higher among nurses who experienced quick return (54.2%) than those who did not (18.9%). Nurses who experienced sleep disturbance experienced more SP (36.9%) than those who did not (16.7%). Additionally, SP was higher among those with health problems (36.9%) than among those without (12.6%). Among non-shift nurses, only total working hours and health problems were significantly associated with SP. The rate of SP was the highest among nurses who worked ≥53 h per week. Nurses who experienced health problems experienced more SP (38.3%) than those who did not (16.4%).

The results of the multivariate logistic regression models are presented in Table 3. The odds of SP among shift nurses who did not have rest breaks during work were approximately four times greater than among those who had breaks (odds ratio, OR = 3.51, 95% CI: 1.17–10.51). Shift nurses who experienced quick return had an approximately five times higher SP risk than those who did not (OR = 8.47, 95% CI: 1.87–38.45). Additionally, the OR of SP was approximately three times greater in shift nurses who experienced sleep disturbance (OR = 3.79, 95% CI: 1.13–12.71) and four times greater in those who had health problems (OR = 4.39, 95% CI: 1.51–12.79) than those who did not. Among non-shift nurses, the OR of SP was approximately eight times greater in nurses who worked ≥53 h per week than those who worked fewer hours (OR = 14.8, 95% CI: 2.31–94.35). 

## 4. Discussion

Employees who work ˃6 h a day are permitted a rest break in European countries [34]. South Korean workers are also required to have a 60 min rest break when they work ˃8 h [32]. However, nurses frequently miss, interrupt, or delay their rest breaks during work [35,36]. Our results indicate that rest breaks during work hours are important for reducing SP in shift nurses. A possible explanation is that rotating shift nurses skip meals more frequently and have fewer breaks compared to non-shift nurses [35]. Only 4% of rotating shift nurses have rest breaks (excluding meal breaks) during work, with an average break time of 15 min, including meal breaks [22]. As the KWCS did not collect data on meal break hours, we could not investigate this further. Future research must consider both meal and rest breaks to confirm the association between breaks and SP. 

Research indicates that increasing the frequency and duration of rest breaks during work improves productivity and reduces SP among workers in other industries [23,37]. For instance, in a study of truck drivers, taking a rest break or a nap reduced the number of driving accidents, which is a clear benefit for both workers and road safety [38]. In the U.S., the Federal Motor Carrier Safety Administration created regulations that truck drivers must take a mandatory 30 min break after eight consecutive hours of driving to ensure safety and reduce driving accidents [39]. Freight truck drivers in South Korea should rest for at least 15 min after two hours of continuous driving to prevent drowsy driving [40]. Rest breaks can help nurses recover from mental and physical strain and positively influence work performance, care quality, and patient safety [36,41]. Therefore, nurse managers and administrators should implement strategies to provide regular rest breaks during work to promote nurses’ health and reduce SP among shift nurses [36,42]. Furthermore, informing nurses about the effects of rest breaks, motivating them to rest, and informing patients about nurses’ breaks are important strategies to create a healthy organizational culture [36,43]. 

Taking adequate rest between shifts was significantly associated with SP among shift nurses. In South Korea, where approximately 70% of nurses work eight-hour rotating shifts, sufficient rest is especially important [44]. Quick return is associated with health hazards such as insomnia, excessive sleepiness, excessive fatigue, and shift work disorder [45]. Moreover, inadequate sleep (or rest) between shifts negatively affects nurses’ performance by increasing the frequency of inattention episodes [46]. Therefore, nurse managers need to implement better work schedule patterns (e.g., rapid and forward rotating shifts) to allow shift nurses adequate time to rest and relieve fatigue before returning to work [45,47,48]. 

Interestingly, working hours were significantly associated with SP only among non-shift nurses. This discrepancy between shift and non-shift nurses might be related to their different working patterns. Generally, in South Korea, non-shift nurses work from Monday to Friday and take two days off on weekends, while rotating shift nurses work two to four consecutive shifts (up to a maximum of five) with fast rotation (i.e., changing shifts from morning to afternoon to night at about two- to three-day intervals). Therefore, inter-shift rest may play a more important role than total working hours for recovery and prevention of SP in shift nurses. Long working hours have been known to increase SP [9], and forced overtime is a significant factor in SP among Swiss hospital employees [15]. Notably, our results also indicate that nurses who work ˃53 h per week are at much greater risk of SP than nurses who work 40 h, which is consistent with the Labor Standards Act limits [32]. Considering the impact of long working hours and overtime on SP, policy development is needed to strictly monitor and penalize hospitals that violate the law related to working hours. 

Sleep plays a significant role in recovery [26]. In particular, nurses who work rotating shifts experience problems like fatigue, decreased awareness, and sleep disturbance [49,50,51]. Research indicates that sleep problems are more prevalent among shift nurses than other populations [52,53]. In line with these results, our study revealed that shift nurses experienced higher rates of sleep disturbance than non-shift nurses (23.2% vs. 15.2%), and shift nurses with sleep disturbance had a greater risk of SP [24,25,26,54]. Sleep length and insomnia predict SP among general employees [25,54]. Sleep quality was negatively related to SP among French nurses and mediated the effect of emotional dissonance and workload on SP [26]. Furthermore, a sleep duration of <8 h is associated with decreased work productivity [24]. Notably, sleep duration may be shortened by insufficient rest periods between work shifts [55], which was a risk factor for increased SP in our study. Therefore, nurse managers should redesign shift schedules considering working hour characteristics, such as shift length, direction and speed of shift rotation, number of consecutive shifts, and rest period between shifts, to avoid sleep disturbance and enhance sleep quality in shift nurses [53,56]. Furthermore, healthcare institutions should have written policies to support the implementation of healthy sleep strategies for nurses [53].

Physical and mental health problems have been associated with SP among general employees and hospital nurses [13,54,57]. Jeon et al. [57] found a positive correlation between chronic health conditions and SP among general workers in South Korea. Additionally, Letvak et al. [13] reported that pain and depression were correlated with SP in American nurses. Nurse managers and administrators should be aware that working with health problems may reduce nurses’ productivity [13], which impacts institutional costs and care quality [58]. Therefore, improving nurses’ health status is crucial to reduce the incidence of SP and increase productivity in hospitals. Regular health examinations for nurses should be provided at the organizational level. Exercise programs, psychoeducation programs, and ergonomic training are suggested to improve nurses’ fitness and mental health and prevent injury [59].

In our study, shift nurses had a higher SP rate than non-shift nurses, which is consistent with evidence concerning European employees [9]. Interestingly, the working hour characteristics and health status factors associated with SP varied between shift and non-shift nurses, suggesting the need for customized strategies for decreasing SP. Breaks during work and between shifts can affect SP among shift nurses. Thus, nurse managers and administrators should implement effective strategies, including redesigning shift schedules, to allow shift nurses opportunities to relieve fatigue and enhance sleep quality. Additionally, more efforts, including regular health examinations, are needed to improve nurses’ health, which is known to be associated with SP. For non-shift nurses, it is necessary to strictly monitor total working hours to reduce the occurrence of SP. 

During the coronavirus disease (COVID-19) pandemic, nurses are expected to work longer hours with more limited breaks. In reality, 33% of nurses in the U.S. worked more than 40 h per week, and 30.5% rarely or never took 30 min breaks while caring for COVID-19 patients [60]. Moreover, they also reported having subthreshold insomnia, chronic and acute fatigue, and psychological health problems during the COVID-19 pandemic [60,61]. To discourage SP and ensure the quality of care, present policies should be reinforced to strictly limit working hours, provide mandatory breaks, and carefully monitor any illegal activities. 

### Limitations

The study has some limitations. First, owing to the use of cross-sectional data, the ability to establish temporal relationships and ascertain causality is limited. Second, the small sample size limits the generalizability of the findings. However, it should be noted that the data were nationally representative. Third, the prevalence of SP was measured using a single item. Despite its use in European studies [15], a single item may not appropriately address the experience of SP. Therefore, studies using reliable and validated tools such as the Stanford Presenteeism Scale, which uses six items to measure SP [62], must be conducted. Fourth, owing to the use of self-reported data, the possibility of recall bias cannot be excluded, and the results may have been under or overestimated. Future research must use objective data such as administrative records concerning work hours and schedules and actigraphy for sleep measurements. Finally, being a secondary data analysis, the study may not have incorporated all the confounding factors that may impact SP in hospital nurses.

## 5. Conclusions

The study’s pre-eminent finding was that breaks during work, quick return, sleep disturbance, and health problems were positively associated with SP among shift nurses, while total working hours were positively associated with SP among non-shift nurses. Nurse managers and administrators should prevent SP in hospital nurses to maintain care quality and enhance productivity. Furthermore, it is necessary to strictly monitor and penalize hospitals that violate the law related to rest breaks during and between shifts and working hours.

## Figures and Tables

**Table 1:** Characteristics of the sample population by shift work (*n* = 272).

Characteristics	Categories	Total	Shift Nurses (*n* = 146)	Non-Shift Nurses (*n* = 126)	x^2^	*p*-Value
		*n* (%)	*n* (%)	*n* (%)		
Age (year)	<30	61(42.5)	40 (45.5)	21 (38.7)	2.34	0.592
	30–39	109 (29.0)	58 (29.6)	51 (28.4)		
	40–49	74 (20.2)	36 (17.4)	38 (23.6)		
	≥50	28 (8.3)	12 (7.5)	16 (9.3)		
Sex	Male	6 (4.8)	4 (4.6)	2 (5.0)	0.02	0.945
	Female	266 (95.2)	142 (95.4)	124 (95.0)		
Education level	Associate degree	80 (30.2)	37 (27.5)	43 (33.6)	2.32	0.342
	Bachelor’s degree	188 (68.5)	106 (70.5)	82 (65.9)		
	Master’s or higher	4 (1.3)	3 (2.0)	1 (0.5)		
Working experience (year)	<1	21 (10.2)	11 (12.4)	10 (7.5)	2.96	0.499
	1–4	121 (55.2)	61 (51.2)	60 (60.2)		
	5–9	71 (18.5)	40 (19.8)	31 (16.9)		
	≥10	59 (16.1)	34 (16.6)	25 (15.4)		
Location	Urban	131 (48.5)	74 (50.8)	57 (45.7)	0.69	0.511
	Rural	141 (51.5)	72 (42.2)	69 (54.3)		
Total working hours	<40	187 (66.8)	107 (70.7)	80 (62.0)	4.39	0.297
	41–52	73 (26.8)	31 (21.9)	42 (32.9)		
	≥53	12 (6.4)	8 (7.4)	4 (5.1)		
Rest break during work	Yes	164 (59.7)	87 (58.9)	77 (60.8)	0.11	0.797
	No	108 (40.3)	59 (41.1)	49 (39.2)		
Quick return	Yes	20 (7.9)	14 (7.2)	6 (8.9)	0.26	0.730
	No	252 (92.1)	132 (92.8)	120 (91.1)		
Sleep disturbance	Yes	62 (19.7)	37 (23.2)	25 (15.2)	2.76	0.144
	No	210 (80.3)	109 (76.8)	101 (84.8)		
Health problem	Yes	99 (33.0)	56 (37.8)	43 (27.0)	3.52	0.108
	No	173 (67.0)	90 (62.2)	83 (73.0)		

Note: Unweighted *n* and weighted percentage are presented.

**Table 2:** Distribution of sickness presenteeism by shift work (*n* = 272).

Characteristics	Categories	Shift Nurses (*n* = 146)				Non-Shift Nurses (*n* = 126)			
		SP	No SP	x^2^	*p*-Value	SP	No SP	x^2^	*p*-Value
		*n* (%)	*n* (%)			*n* (%)	*n* (%)		
Age (year)	<30	7 (15.4)	33 (84.6)	8.87	0.240	5 (25.2)	16 (74.8)	4.57	0.626
	30–39	18 (32.4)	40 (67.6)			12 (20.9)	39 (79.1)		
	40–49	7 (17.7)	29 (82.3)			6 (14.9)	32 (85.1)		
	≥50	3 (22.7)	9 (77.3)			5 (33.7)	11 (66.3)		
Sex	Male	0 (0.0)	4 (100.0)	3.60	0.345	1 (10.9)	1 (89.1)	1.08	0.525
	Female	35 (22.4)	107 (77.6)			27 (22.9)	97 (77.1)		
Education level	Associate degree	10 (20.2)	27 (79.8)	11.18	0.062	10 (18.9)	33 (81.1)	1.37	0.700
	Bachelor’s degree	23 (20.2)	83 (79.8)			18 (24.3)	64 (75.7)		
	Master’s or higher	2 (79.0)	1 (21.0)			0 (0.0)	1 (100.0)		
Working experience (year)	<1	1 (9.4)	10 (90.6)	15.50	0.094	2 (5.9)	8 (94.1)	5.55	0.406
	1–5	13 (19.3)	48 (80.7)			13 (22.9)	47 (77.1)		
	5–10	15 (40.0)	25 (60.0)			8 (31.0)	23 (69.0)		
	≥10	6 (14.7)	28 (85.3)			5 (18.6)	20 (81.4)		
Location	Urban	15 (20.2)	59 (79.8)	0.23	0.762	18 (32.5)	39 (67.5)	13.63	0.057
	Rural	20 (22.6)	52 (77.4)			10 (13.8)	59 (86.2)		
Total working hours	<40	25 (22.8)	82 (77.2)	1.24	0.726	16 (14.5)	64 (85.5)	26.48	0.027 *
	41–52	8 (19.9)	23 (80.1)			10 (29.8)	32 (70.2)		
	≥53	2 (12.5)	6 (87.5)			2 (69.2)	2 (30.8)		
Rest break during work	Yes	14 (15.7)	73 (84.3)	7.47	0.086	14 (15.9)	63 (84.1)	9.97	0.099
	No	21 (29.5)	38 (70.5)			14 (32.3)	35 (67.7)		
Quick return	Yes	7 (54.2)	7 (45.8)	13.47	0.008 **	0 (0.0)	6 (100.0)	7.61	0.243
	No	28 (18.9)	104 (81.1)			28 (25.5)	92 (75.5)		
Sleep disturbance	Yes	15 (36.9)	22 (63.1)	11.78	0.029 *	9 (41.3)	16 (58.7)	10.08	0.074
	No	20 (16.7)	89 (83.3)			19 (19.0)	82 (81.0)		
Health problem	Yes	23 (36.9)	33 (64.1)	20.62	0.005 **	17 (38.3)	26 (61.7)	14.74	0.039 *
	No	12 (12.6)	78 (87.4)			11 (16.4)	72 (83.6)		

Note: Unweighted *n* and weighted percentage are presented. SP = sickness presenteeism. * *p* < 0.05, ** *p* < 0.01.

**Table 3:** The odds ratios of working hour characteristics and health status on sickness presenteeism (*n* = 272).

Characteristics	Categories	Shift Nurses		Non-Shift Nurses	
		OR	95% CI	OR	95% CI
Total working hours	<40	1.00	reference	1.00	reference
	41–52	0.65	0.19–2.23	3.03	0.92–9.94
	≥53	0.19	0.03–1.15	14.8	2.31–94.35
Rest break during work	Yes	1.00	reference	1.00	reference
	No	3.51	1.17–10.51	1.40	0.46–4.22
Quick return	Yes	8.47	1.87–38.45	-	-
	No	1.00	reference	-	-
Sleep disturbance	Yes	3.79	1.13–12.71	3.01	0.90–9.99
	No	1.00	reference	1.00	reference
Health problem	Yes	4.39	1.51–12.79	2.65	0.92–7.60
	No	1.00	reference	1.00	reference

Note: OR = odds ratio; CI = confidence interval. Values are adjusted for age, educational level, work experience, and location. Quick return was excluded in the model of non-shift nurses due to multicollinearity.

## Data Availability

Publicly available datasets were analyzed in this study. These data can be found here: The 5th Korean Working Conditions Survey. Available online: https://kosha.or.kr/kosha/data/primitiveData.do (accessed on 19 March 2021).
